# Role of NLRs in the Regulation of Type I Interferon Signaling, Host Defense and Tolerance to Inflammation

**DOI:** 10.3390/ijms22031301

**Published:** 2021-01-28

**Authors:** Ioannis Kienes, Tanja Weidl, Nora Mirza, Mathias Chamaillard, Thomas A. Kufer

**Affiliations:** 1 Department of Immunology, Institute for Nutritional Medicine, University of Hohenheim, 70599 Stuttgart, Germany; ioannis.kienes@uni-hohenheim.de (I.K.); tanja.weidl@uni-hohenheim.de (T.W.); Nora_Mirza@uni-hohenheim.de (N.M.); 2 University of Lille, Inserm, U1003, F-59000 Lille, France; mathias.chamaillard@inserm.fr

**Keywords:** NOD-like receptors, Interferons, innate immunity, immune regulation, type I interferon, antiviral, signaling

## Abstract

Type I interferon signaling contributes to the development of innate and adaptive immune responses to either viruses, fungi, or bacteria. However, amplitude and timing of the interferon response is of utmost importance for preventing an underwhelming outcome, or tissue damage. While several pathogens evolved strategies for disturbing the quality of interferon signaling, there is growing evidence that this pathway can be regulated by several members of the Nod-like receptor (NLR) family, although the precise mechanism for most of these remains elusive. NLRs consist of a family of about 20 proteins in mammals, which are capable of sensing microbial products as well as endogenous signals related to tissue injury. Here we provide an overview of our current understanding of the function of those NLRs in type I interferon responses with a focus on viral infections. We discuss how NLR-mediated type I interferon regulation can influence the development of auto-immunity and the immune response to infection.

## 1. Type I Interferons

Interferons (IFNs) are a heterogenous group of proteins that can be classified into three families (Type I, II, and III) based on distinct functions and characteristics [1]. The family of human type I IFN is composed of 5 subgroups: IFN-α, -β, -κ, -ε, and -ω [2,3,4], whereas the type II IFN group only contains IFN-γ [3]. Type III IFNs are composed of four IFN-λ proteins [5,6]. 

This review will focus on the regulation of type I IFNs by members of the Nod-like receptor (NLR) family, and within this class on the most prominent and best studied members IFN-α and IFN-β. 

**Figure 1:**
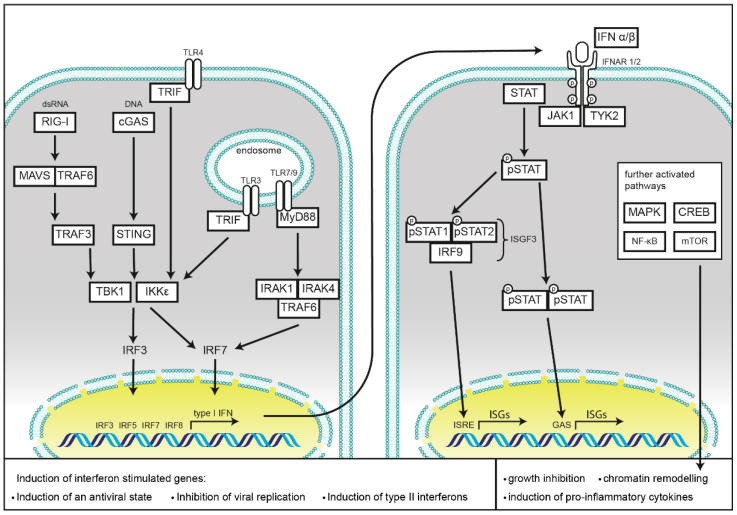
Schematic representation of cellular type I interferon secretion. Induction pathways and the main components of interferon production (left) and type I interferon signaling (right) are shown.

Type I IFNs all bind to a common heterodimeric receptor consisting of the IFN-α/β R1 (IFNAR1) and IFN-α/β R2 (IFNAR2) subunits [7,8,9], which are expressed on most cell types. Binding of type I IFNs to their receptor causes receptor subunit dimerization [10], rapid activation of the R2 subunit associated Janus kinase 1 (JAK1) [11,12], and subsequently induction of the JAK-STAT pathway [13]. This tyrosine kinase auto-phosphorylates and additionally phosphorylates specific residues within the interaction sites of the intracellular domain of the receptor, revealing signal transducer and activator of transcription (STAT) binding pockets [14]. After binding of the STAT proteins via their Src-homology 2 (SH2) domains, STATs get phosphorylated by activated JAK1, leading to their dissociation from the receptor. IFN-α induces the formation of STAT1/STAT2 heterodimers [15], which can further associate with interferon regulatory factor 9 (IRF9), and subsequently form the IFN-stimulated gene factor 3 (ISGF3) [16]. The ISGF3 translocates into the nucleus to bind interferon stimulated response elements (ISREs), inducing antiviral response genes [15,17,18]. Furthermore, STAT1 can form homodimers or heterodimers with STAT3. STAT1, STAT3, STAT4, STAT5, and STAT6 form homodimers. Dimerization precedes translocation into the nucleus and activation of genes regulated by a gamma interferon activation site (GAS) [19,20,21], causing a pro-inflammatory response (Figure 1). 

Binding of IFN-α to its receptor also leads to rapid phosphorylation of receptor subunit R1 associated tyrosine kinase Tyk2 [22,23,24,25], which mediates signaling to non-IFN pathways. This results in initiation of the MAP kinase pathway, activation of p38 and subsequent growth inhibition [26], as well as chromatin remodeling upon translocation of the Cre binding element (CREB) [27]. Furthermore, Tyk2 activates phosphoinositide-3-kinase (PI3-K), resulting in the activation of the mammalian target of rapamycin (mTOR) pathway and initiation of mRNA translation, as well as activation of the pro-inflammatory nuclear factor ‘kappa-light-chain-enhancer’ of activated B-cells (NF-κB) pathway [28].

### 1.1. Immune Response to Infection and Tissue Tolerance are Influenced by the Type I Interferon Response

Viruses interact with a broad range of proteins in mammalian cells, and their evolution has been driven by antiviral constraints and adaptation of their host cells. It is hence not surprising that their co-evolution has resulted in highly sophisticated regulatory mechanisms of the timing and amplitude of immune responses to viral challenges. Type I IFNs have a central role in controlling viral infections and are also involved in the defense of other pathogens. In 1957, IFNs were discovered by Alick Isaacs and Jean Lindenmann, as a soluble factor in the supernatant of chorio-allantoic membrane, challenged with heat inactivated influenza virus, that interferes with the viral infection in cells, hence the name “interferon” [29]. Type I IFNs act both in an autocrine and paracrine manner, and prime bystander cells for upcoming viral infection by the latter. Their ability to restrict viral replication is mainly driven by a multitude of interferon-stimulated genes (ISGs). Furthermore, type I IFNs play an important role in the activation of cells that are involved in the development of the adaptive immune response. Here type I IFNs take part in the control of cell expansion and differentiation and determining cytokine and chemokine responses of cells of the lymphoid lineage [30].

Type I IFNs are associated with the rapid induction of a cellular antiviral state, and most cells can produce them in response to an appropriate pattern-recognition receptor (PRR) stimulation. They prime the infected cells, as well as the surrounding cells towards a state of either defense or tolerance [31]. Their importance as protective factors during viral infections was proven by showing the high susceptibility of mice deficient in the IFNAR1 receptor (*Ifnar1^−/−^* mice) to vesicular stomatitis virus (VSV), Semliki Forest virus, vaccinia virus (VACV), and lymphocytic choriomeningitis virus (LCMV) [32]. Furthermore, mice with STAT1 deficiency were shown to be highly susceptible to influenza viruses [33], further cementing the importance for type I IFNs in antiviral responses. In humans, several forms of inherited STAT1 deficiencies are associated with a high susceptibility to intracellular bacteria and viruses [34], while some gain-of-function STAT1 mutations are responsible for the development of chronic mucocutaneous candidiasis [35].

In bacterial infections, the functions of type I IFNs are more complex, as they can influence host defense either positively or negatively [30]. Type I IFN treatment of macrophages results in better restriction of bacterial replication during infection with intracellular *Legionella pneumophilia* or *Bacillus anthracis* [36,37,38,39]. Furthermore, type I IFN appears to protect cells from invasion by *Salmonella enterica subsp. enterica ser. Typhimurium* (*S. Thphimurium)* and *Shigella flexneri*, as mice treated with recombinant type I IFN showed reduced numbers of invasive bacteria in epithelial cells and improved survival [40,41]. Type I IFNs contribute to the activation of macrophages, regarding production of nitric oxide (NO) and TNFα [42]. However, IFN-α and -β have also been identified as negative regulators of many of the cytokines and chemokines, orchestrating immune responses to bacterial infections, in particular for *Listeria monocytogenes* [43,44] and *S. Thphimurium* [44,45] (reviewed in [46]). 

Besides bacteria, recognition of fungi, most importantly by the C-type lectin receptor Dectin-1, but also of fungal nucleic acids by Toll-like receptor 7 (TLR7) and TLR9 induces robust type I interferon responses [47,48]. However, as with bacterial infections, type I interferons can also be supportive for pathogen survival [49]. 

Type I IFNs are of equal importance in orchestrating adaptive immune responses to infection by transcriptional regulation of a broad range of target genes. Notably, type I IFNs induce and support the production of type II IFNs, mainly IFN-γ in NK cells directly [50,51], and support production of IL-12 in dendritic cells (DCs) [52]. They can further enhance responses of myeloid cells, B cells, and T cells upon viral infection, leading to improved clearance of viruses and to establishment of a robust adaptive T and B cell memory repertoire. In antigen presentation, IFN-γ induces the transcription of MHC class I and class II by inducing the expression of two NLR family members, caspase activation and recruitment domain (CARD) containing 5 (NLRC5) and MHC class II transcriptional activator (CIITA), respectively [53,54]. Meanwhile it was found that the expression of many other NLRs is regulated by both type I and type II IFNs. In the following section, we describe in detail how NLRs are regulated by type I IFNs and how they modulate the outcome of type I IFN responses. We discuss how deregulation of NLRs can result in susceptibility to either infection or auto-inflammatory disease as a consequence of pathogen dissemination or a lowered tissue tolerance to stress damage. 

### 1.2. Induction of Type I Interferon Response by Nucleic Acid Sensing 

Recognition of pathogen-associated molecular patterns (PAMPs) by evolutionary conserved PRRs is the initial step for mounting of a rapid innate immune response. After sensing potentially noxious non-self molecules, PRRs activate a defined set of signaling cascades, culminating in induction of a state of tolerance or defense in the host cell. This allows the production and release of cytokines, which signal to neighboring cells for recruiting immune cells for the initiation of a specific adaptive immune response. 

PRRs are localized in different subcellular compartments. Toll-like receptors (TLRs), C-type lectins, and scavenger receptors cover the cell surface, as well as, in the case of TLRs, membranes of the endosomal compartment. NOD-like receptors (NLRs), RIG-I like receptors (RLRs), and cyclic GMP-AMP synthase (cGAS) monitor the cytoplasm for cell damage or the presence of invasive pathogens. Activation of these receptors results in the induction or repression of type I IFNs secretion, which will be discussed in the following chapters and is summarized in Figure 1.

Detection of cytosolic DNA is mainly mediated by the ubiquitously expressed cGAS and the absent in melanoma 2 (AIM2) protein. This not only includes foreign DNA derived from pathogens, but also cytosolic chromatin resulting from genotoxic stress. While cGAS activation induces type I IFNs, detection of cytosolic DNA by AIM2 results in pyroptotic cell death as a consequence of the activation of caspase-1 and the subsequent processing and release of mature IL-1β and IL-18 [55]. Binding to cytosolic DNA renders cGAS in an active state, leading to the synthesis of the second messenger cyclic GMP-AMP (cGAMP) with a mixed-linkage backbone (c[G(2′,5′)pA(3′,5′)p]), which in turn is sensed by the protein referred as stimulator of interferon genes (STING) [56,57,58,59], located at the membrane of the endoplasmic reticulum [60]. Activation of STING leads to its translocation into the Golgi network and activates the TRAF family member associated NF-κB activator-binding kinase 1 (TBK1). After auto-phosphorylation, TBK1 subsequently activates IRF3 through direct binding [61]. This enables its dimerization, translocation into the nucleus, and initiation of transcription of type I IFNs. IRF3 activation results in an initial wave of transcription with IFN-β and IFN-α4 as central transcription targets. Transcription of IRF7 is also induced for allowing a positive feedback loop leading to a second wave of type I IFNs secretion [62]. STING is the essential mediator of this response as its deficiency abolishes cGAS-induced IRF3 activation and IFN-β induction [63]. cGAS deficiency in mouse bone marrow-derived macrophages (BMDMs) has been shown to be detrimental to induction of antiviral type I IFN responses towards DNA viruses such as herpes simplex virus (HSV) 1, VACV, and murine gammaherpesvirus 68, but does not influence the response towards the RNA virus Sendai virus (SeV) [64,65]. Besides activation of IRF3, STING also functions as an activator of NF-κB. For an extensive review on the functions of cGAS-STING activation, the reader is referred to [66]. 

Studies in cells from *cGAS*^−/−^ mice have proven that cGAS is the main DNA sensor in antigen presenting cells, such as plasmacytoid dendritic cells (pDCs) and conventional dendritic cells (cDCs). Depletion of cGAS in those cells rendered them unresponsive towards DNA transfection and infection with DNA viruses [67]. The type I IFN response towards these nucleic acids is also essential as a priming signal for the function of the DNA-induced AIM2 inflammasome assembly [55]. 

Besides nucleic acids from several DNA viruses like cytomegalovirus [68,69], HSV 1 [67], VACV [67], and retroviruses [70], cGAS is also the sensor for microbial DNA from invasive bacteria and protozoans such as *L. monocytogenes* [71,72,73], *Chlamydia trachomatis* [74], *Mycobacterium tuberculosis* [75,76,77], *Toxoplasma gondii* [78], and *Leishmania major* [79].

The most important family of cytosolic RNA-sensors is the RIG-I-like receptor family (RLRs), consisting of the retinoic acid-inducible gene I protein (RIG-I), melanoma differentiation-associated protein 5 (MDA5), and laboratory of genetics and physiology 2 (LGP2). These proteins are able to sense the 5-prime di- and tri-phosphates of short, blunt end double stranded (ds)RNA by RIG-I, or long dsRNA by MDA5 [80]. All three proteins contain DExD/H box domains with ATPase function, which are crucial for RNA binding. RIG-I and MDA5 further contain two CARD. These N-terminal domains are responsible for further downstream signaling by binding to the CARD domain of the mitochondrial antiviral signaling protein (MAVS). The C-terminal domain of RIG-I serves as an inhibitory domain, keeping the protein in an inactive state until it binds RNA and conformational changes are induced [81]. 

After the binding of different cytosolic RNA species, both MDA5 and RIG-I are subject to K63-linked ubiquitination, both by covalent, and non-covalent attachment [82]. Either RIG-I, tripartite motif-containing protein 25 (TRIM25) [82], or Riplet [83,84] can function as E3 ubiquitin ligases. This process enables RIG-I to homotetramerize [85] and localize to MAVS at the outer mitochondrial membrane initiating its oligomerization [86]. This multimerization of MAVS results in its activation and enables recruitment of additional downstream adaptor proteins TRAF2, TRAF6, and TRADD [87,88]. Subsequently TRAF3 [89] and TANK [90] are recruited to facilitate the activation of TBK1 and IKKε, which then phosphorylate the transcription factors IRF3 and IRF7. Activation of those two factors enables their homodimerization and translocation into the nucleus where they initiate transcription of type I and type III IFNs [91,92,93,94]. LGP2 does not contain a CARD domain, and hence was proposed not to function in signaling, but rather as a regulator of RIG-I or MDA5 function [95]. 

### 1.3. Induction of Type I Interferon Responses by Membrane Bound TLRs 

While most of the members of the TLR family of TLRs may activate NF-κB signaling cascade by MyD88, type I IFNs are induced by TLRs via activation of TRIF [96]. Among those TLRs, TLR4 has proven to be the most important inducer of type I IFNs. Recognition of LPS, or several viral proteins, leads to the activation of TRIF. TRIF can then directly associate with TBK1, inducing IRF3 activation and translocation into the nucleus as described above [97,98]. Further, TLR3, which also signals via TRIF, and TLR7 and TLR9 are inducers of IFN responses [98]. TLR7 and TLR9, are mainly expressed in pDCs where they induce type I IFN expression in a MyD88-dependent manner. pDCs constitutively express IRF7, and it has been shown that MyD88 can form a complex with IRF7 to trigger its activation and transcriptional activity [99,100]. For a more comprehensive review on TLR induced immune signaling, see [101,102]. 

### 1.4. Induction of Interferon Responses by NLRs

Besides membrane-bound TLRs and cytosolic RLRs, the NOD-like receptor (NLR) protein family is another group of cytosolic PRRs. In mammals, a total of 22 human NLRs have been described [103]. NLRs are characterized by a common tripartite motif, consisting of a central nucleotide binding and oligomerization (NACHT) domain, C-terminal leucine rich repeats (LRRs) and a variable N-terminal effector domain. According to their effector domain, NLRs are categorized into different subgroups: CARD-transcription and activation domain (CARD-AD) containing NLRA, baculovirus inhibitor of apoptosis (BIR) domain carrying NLRB, caspase activation and recruitment domain (CARD) containing NLRC and pyrin domain (PYD) containing NLRP [104]. NLRX1 contains an unconventional N-terminal domain, which shares no homology with the N-terminal domains of the other protein-family members. It is further unique as it contains a mitochondrial localization sequence (MLS) [105]. 

NOD1 and NOD2 were the founding and name giving members of this protein family [106,107,108]. NOD1 and NOD2 function as intracellular sensors of peptidoglycan (PGN) components from the bacterial cell wall to initiate an appropriate immune response [106,107,109,110,111]. However, not all proteins of this subfamily function as bona fide PRRs. This is indicated by the fact that no direct ligand binding, or even direct activator, has been discovered for most members of the NLR protein family. Furthermore, some of the NLRs with known activators, like NLRC4 [112], do not bind to their activators directly, but rather need accessory proteins. Besides the function of NLRs as PRRs with direct induction of pro-inflammatory signaling pathways (NOD1, NOD2, NLR family apoptosis inhibitory protein NAIP), some NLRs form a specialized multiprotein complex, the inflammasome. Inflammasome formation has in common, that apoptosis associated speck like protein (ASC), is recruited by PYD of the activated NLR. Consequently, a highly organized multiprotein signaling platform is built, to which pro-caspase-1 is recruited, resulting in the maturation of pro-IL-1β and pro-IL-18 [113]. Non-PRR functions have also been described for two other NLR proteins, namely MHC class II transactivator (CIITA) and NLRC5, which are transcriptional regulators, that have been described to shuttle into the nucleus, where they can interact with a multiprotein transcription complex, termed MHC enhanceosome, to induce the transcription of MHC class II and MHC class I genes, respectively [114,115,116,117]. Nuclear translocation and direct transcriptional regulation have further been described for NLRP3 [118] and NOD2 [119]. Several other NLRs have been recently described as modulators of innate immune responses. For details about the functions of NLR proteins, the reader is referred to recent review articles [120,121,122]. However, to this date, there are still several NLR proteins whose functions have not been studied. 

**Figure 2:**
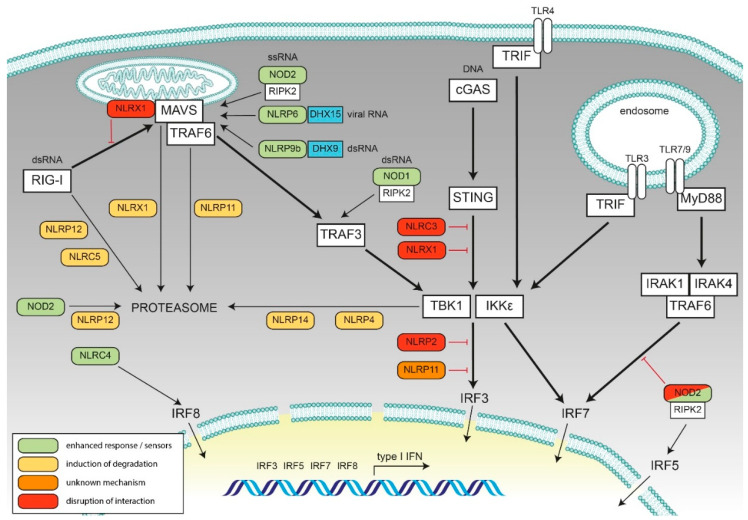
Schematic representation of the role of the discussed NLRs in type I interferon signaling. The key viral sensing pathway components leading to type I interferon production are shown in the white boxes, and the interferon signal transduction pathways are depicted in bold lines in a schematically depicted cell. Cooperation and interference of NLRs are shown. Green: NLRs with enhancing activity, yellow: NLRs leading to reduced interferon (IFN) response due to degradation of key components, red: Inhibition of signaling by steric hindrance, orange: Negative regulation by unknown mechanism. Cooperating factors for viral RNA sensing by NLRs are shown in blue.

In the following sections, we provide an overview of our current understanding of the functions of NLRs in IFN responses. For a summary see Table 1 and Figure 2.

## 2. Negative Regulatory Feedback on Type I Interferon Responses by NLRs

### 2.1. NLRX1

NLRX1 has been associated with diverse signaling pathways. It attenuates NF-κB activation upon TLR activation [138,139,176] and can enhance ROS production, thereby enhancing the JNK pathway [177,178,179,180]. Furthermore, NLRX1 also promotes autophagy through association with Tu translation elongation factor (TUFM) [140] and enhances IRF1 protein levels upon viral infection by attenuating the inhibition of mRNA translation by protein kinase R (PKR) [181]. NLRX1 has also been implicated in the induction of apoptosis [182] and regulation of the NLRP3 inflammasome [183,184].

Besides those functions, NLRX1 is one of the best described NLRs that regulates type I IFN responses. NLRX1 does not seem to be a sensor of viral or bacterial infection, but rather a negative regulator of type I IFNs [105,138]. Its unusual function is underscored by the fact that it contains a MLS in its N-terminus [105,178,185]. Although, the exact localization at the mitochondria is still a matter of debate, as both localization to the mitochondrial matrix and to the outer mitochondrial membrane [105] have been reported.

Through interaction with MAVS, NLRX1 negatively regulates RIG-I-MAVS-dependent IFN-β induction by disruption of the interaction of MAVS and RIG-I [105,138]. Hence, overexpression of NLRX1 results in impaired RIG-I-dependent antiviral signaling and thereby enhanced viral replication [141,142]. NLRX1 might target MAVS for proteasomal degradation through recruitment of poly (rC) binding protein 2 (PCBP2) which is recruited by the NACHT domain of NLRX1 [142]. Silencing of NLRX1 in pDCs, where NLRX1 is constitutively expressed, and in monocyte derived DCs (moDCs), in which basal levels of NLRX1 are increased during differentiation, also leads to higher RLR-induced levels of type I IFN [143], supporting a negative regulation of RIG-I-induced signaling.

Knockdown of NLRX1 leads to enhanced transcription levels of *IFNb1*, *STAT2*, and the 2′-5′-oligoadenylate synthetase 1 gene (*OAS1*) after viral infection, suggesting a negative regulatory role of NLRX1 on the IFN-β/STAT2/OAS1 axis [138]. Accordingly, virus infection causes higher expression of *IFNa2*, *IFNb1*, *OAS1*, and *STAT2* in *Nlrx1^−/−^* mice when compared to wild-type mice. However, such heightened antiviral response lowered tissue tolerance towards lung damage [138]. Fas-associated factor 1 (FAF1) on the other hand, was identified as an inhibitor of NLRX1-mediated reduction of type I IFN expression. FAF1 competes with MAVS for binding to NLRX1 and therefore positively regulates virus-induced type I IFN secretion. It is proposed that upon FAF1 binding, NLRX1 dissociates from MAVS, which is then able to interact with RIG-I and enhance type I IFN induction [144]. Another mechanism through which NLRX1 can inhibit the induction of type I IFNs is by binding to STING. This interaction is enhanced upon viral infection and dissociates TBK1 from the protein complex [145]. Furthermore, NLRX1 is involved in the regulation of autophagy. Interaction of mitochondrial TUFM with NLRX1 was suggested to enhance autophagy and by that to inhibit type I IFN signaling [140].

It should be noted that the inhibitory effect of NLRX1 on MAVS-dependent type I IFN induction is somewhat controversial, as several groups could not validate the above-described effects [146,147,148]. As it was shown that NLRX1 differentially affects IRF3- and IRF1-mediated responses, this might explain, at least in part, these contradictory findings [181].

### 2.2. NLRC3

NLRC3 can negatively regulate several signaling pathways such as NF-κB [186,187], mTOR [188], and the assembly and activity of the NLRP3 inflammasome [189]. It was further shown to attenuate auto-immune and virus specific CD4^+^ T cell responses by inhibition of TNF and IFN-γ production [187,190] and by that reducing proliferation of T_h_1 and T_h_17 cells [187]. 

NLRC3 also restricts type I IFN production in response to cytosolic DNA, cyclic di-GMP (c-di-GMP), and HSV1 infection by directly impeding the interaction between STING and TBK1 [149]. Mechanistically, NLRC3 blocks STING trafficking from the ER to a perinuclear/golgi location and to endoplasmic-associated puncta after DNA sensing [149]. This negative regulation of STING by NLRC3 prevents TBK1-dependent phosphorylation of IRF3 through its binding to Ras GTPase-activating-like protein IQGAP1 [191]. NLRC3 deficiency in murine BMDMs and MEFs results in higher DNA- and HSV1-induced type I IFN, IL-6, and TNF production. Consequently, *Nlrc3^−/−^* mice infected with HSV1 show reduced morbidity and viral load [149]. NLRC3 might also play a role in RIG-I-induced IFN response [192], however, the predominant effect is on the cGAS-induced pathway [149]. 

Negative regulation of TLR signaling by NLRC3 is mediated by formation of a complex with TRAF6, and it was proposed that cellular complexes of TRAFs with regulatory NLRs, dubbed “TRAFasomes”, exist that act as regulatory platforms [186]. It remains to be established if such a scenario might also contribute to the regulation of interferon pathways by NLRC3.

Beside its function as a negative regulator, NLRC3 can bind double-stranded viral DNA by its LRR with high affinity, which leads to an increase of its ATPase activity of the NBD by 10-fold. The binding of ATP diminishes the interaction of the NBD with STING, leading to the activation of the type I IFN pathway [193]. 

### 2.3. NLRC5

NLRC5 is part of a distinct set of NLRs that function as transcriptional regulators of MHC class I and class II genes [114,151,194]. Both NLRC5 and CIITA bind to their respective transcriptional targets in MHC promotor regions via the same multiprotein DNA binding complex [115,117,195,196]. NLRC5 is constitutively expressed in a broad range of lymphoid organs and barrier tissues, such as the lung and the gastrointestinal tract, that are a gateway for several pathogens [114,151,194]. Expression of NLRC5 and subsequent MHC class I gene expression can be enhanced by stimulation with IFN-γ [114,151,194,197].

In the first characterization of NLRC5, it was reported that it influences transcription from ISRE and GAS reporter elements, while overexpression of NLRC5 resulted in elevated levels of IFN-α mRNA in HeLaS3 cells. Those results were confirmed by siRNA-mediated knockdown [114], and we showed that in THP-1 cells and primary dermal fibroblasts siRNA knockdown of NLRC5 reduces IFN-β and CXCL10 induction upon SeV infection [151]. NLRC5 was shown to inhibit influenza A virus (IAV) replication in the lung epithelial cell line A549 and to increase RIG-I and type I IFN transcription [152]. Interaction between NLRC5 and RIG-I was confirmed independently by Cui et al. However, these authors reported a negative effect of NLRC5 overexpression on type I IFN luciferase reporter activation by poly(I:C) [153]. Meanwhile, knockdown of NLRC5 in several different cell lines was shown to lead to increased IFN-β responses towards either poly(I:C) treatment or VSV infection [153]. However, the regulation of IFN-β activation by NLRC5 remains a matter of debate [151]. Noteworthy, *Nlrc5^−/−^* mice in which exon 4 was targeted neither showed altered basal nor poly(I:C)-induced IFN-β serum levels compared to wildtype animals [154]. This is contrasted by studies using another NLRC5 knockout mouse model, in which exon 8 was targeted. Ex vivo stimulation with VSV or poly(I:C), as well as systemic challenge with VSV resulted in higher levels of IFN-β and stronger phosphorylation of IRF3 [155]. 

While the role of NLRC5 as a key regulator of MHC class I gene regulation is well established, the role of NLRC5 in type I IFN responses seems to be highly dependent on the cell type and the organismic context [156]. This is well illustrated by the observation, that knockdown of NLRC5 increases RIG-I-induced antiviral IFN response in pDCs, while it does not affect the same pathway in moDCs. Interestingly, those two cell types differ in their basal expression level of NLRC5 [143], suggesting a differential contribution of NLRC5 to IFN control in these cell types. Besides its role in antigen presentation, it is plausible that NLRC5 has further roles in antiviral immunity linked to the innate detection of viruses beside its role in antigen presentation, as suggested by some of the studies discussed above. 

### 2.4. NLRP2

In humans, NLRP2 is predominantly expressed in the brain, pancreas, kidney, and reproductive tissues such as testis and placenta [157,198,199]. In immune cells, NLRP2 is upregulated in macrophages in response to the B-DNA analog dAdT, as well as in T cells upon activation of RIG-I [198]. Differences exist between mouse and human cell populations. In contrast to human cells, NLRP2 is not upregulated in mouse CD3^+^ T cells upon RNA and DNA sensing, while in mouse CD14^+^ myeloid cells, RNA sensing leads to increased expression of NLRP2 [198]. NLRP2 protein levels were shown to be upregulated upon IFN-β, IFN-γ, and LPS treatment in macrophage like differentiated human THP-1 cells, whereas CpG treatment did not affect NLRP2 protein levels [157]. Among the cytokines that are regulating NLRP2 expression, co-treatment with IFN-γ and TNF-α enables non-canonical inflammasome activation by intracellular LPS in brain pericytes [158]. 

In terms of type I IFN regulation, NLRP2 can bind TBK1 leading to perturbed interaction with IRF3, resulting in reduced IFN-β production [159], albeit this is a singular observation at present.

### 2.5. NLRP4

NLRP4 has only been studied recently. Although containing a PYD, NLRP4 does not interact with the inflammasome adaptor protein ASC [200] and has no effect on IL-1β secretion [201]. It was described to regulate formation of the autophagosome and autophagic processes [201,202], and to negatively regulate the NF-κB response [163,203]. Furthermore, NLRP4 has been described to play a role in embryonic development [204]. It is expressed in human oocytes and early embryos [205], and seven gene copies of Nlrp4 are expressed in murine oocytes [206,207,208]. Knockdown of Nlrp4e in murine oocytes causes developmental arrest between the 2- and the 8-cell stage [204].

NLRP4 represses type I IFN responses by targeting TBK1 for degradation. This is mediated by recruitment of the deltex E3 ubiquitin ligase 4 (DTX4). NLRP4 interacts with the kinase domain of phosphorylated TBK1 which facilitates K48-linked polyubiquitination of TBK1 at lysine residue 670 by DTX4 [164]. This degradation might be mediated by a signalosome complex including NLRP4, ubiquitin specific peptidase 38 (USP38), DTX4, TRAF interacting protein (TRIP), and potentially some phosphatases that remain to be identified. Upon viral infection, TBK1 gets activated, resulting in its K63- and K33-linked ubiquitination. The formation of this complex leads to editing of K33-linked ubiquitination at lysine residue 670 at TBK1 and its replacement by K48-linked polyubiquitination [165]. However, this might not be the only pathway as the dual specificity tyrosine phosphorylation regulated kinase 2 (DYRK2) was found to contribute to NLRP4-mediated degradation of TBK1 [166]. DYRK2 phosphorylates TBK1 on serine residue 527, which is essential for the recruitment of NLRP4 and enhances the interaction of the two proteins. This promotes K48-linked polyubiquitination of TBK1. The authors suggest that DYRK2 enhances the degradation of TBK1 through the NLRP4-DTX4 nexus [166]. In rat cardiac muscle cells, reduced TBK1 and IRF3 levels were reported upon overexpression of NLRP4 in a dose-dependent manner [163]. 

We still know very little about the physiological function of NLRP4. The association of knockdown of NLRP4 isoforms with developmental defect in oocytes might result from missing tolerance towards paternal DNA as has been shown for NLRP14 (see below). Data cumulating from the studies summarized above suggest that a key mechanism of NLRP4 is the control of the half-life of TBK1 through proteasomal degradation. 

### 2.6. NLRP11

The most recent addition to the list of NLRP proteins with regulatory effects on type I IFN signaling is NLRP11. Its gene is located in a gene cluster together with NLRP8 and NLRP13, and lies antiparallel adjacent to the known regulator of type I IFN responses NLRP4. It was hence speculated that NLRP11 might share functional similarity with NLRP4. Indeed, NLRP11 recently has been identified as a negative regulator of the type I IFN response. Overexpression of NLRP11 has been shown to inhibit SeV and poly(I:C) induced IFN-β responses in THP-1 cells [170,171]. Qin et al. proposed binding of NLRP11 to the MAVS-coupled signalosome and subsequent destabilization of the adaptor protein TRAF6 as the regulatory mechanism [171], however, we showed that NLRP11 also inhibits TBK1-induced type I IFN responses, arguing for an alternative mechanism, acting downstream of the activation of TRAF6 [170]. We still know little about the physiological role of NLRP11, and a homolog of NLRP11 seems to be absent in mice [209]. The high expression of NLRP11 in B cells [170] might be suggestive of a role of NLRP11 in B cell tropic viral infections.

### 2.7. NLRP12

NLRP12 is another regulatory NLR protein with multiple functions in bacterial, parasitic, and viral immune responses. It can form an inflammasome [210] that is activated upon infection with bacteria such as *Yersinia pestis* [211] or protozoans like *Plasmodium* [212]. In contrast, reduced NLRP12 levels correlate with higher levels of IL-1β in a high-fat diet model of obesity [213], and Silveira et al. observed an increased production of IL-1β and caspase-1 activity in *Brucella abortus* infected murine BMDM from *Nlrp12^−/−^* animals [214]. Besides its role in inflammasome signaling, NLRP12 possesses anti-inflammatory functions in different pathways. It inhibits MAPK signaling and negatively regulates the canonical as well as non-canonical NF-κB pathway [214,215,216,217]. In humans, the importance of NLRP12 as an anti-inflammatory regulator is underlined by the observation that rare mutations in NLRP12 are causing familial cold auto-inflammatory syndrome (FCAS) [218]. 

NLRP12 has been proposed to function as a myeloid checkpoint for IFN induction by RNA viruses. In response to either VSV infection or the RIG-I agonist 5′ppp-dsRNA and short poly(I:C), NLRP12 deficiency in human and mouse DCs results in increased production of IFN-β and its related cytokines, including TNF [173]. In vivo, *Nlrp12^−/−^* mice show an increased type I IFN and TNF response to VSV infection, which results in lower viral load and faster recovery [173]. Mechanistically, NLRP12 attenuates RIG-I mediated immune signaling by promoting RNF125-mediated degradation of RIG-I. It further enhances K63-linked ubiquitination of RIG-I by TRIM25, preventing its activation [173]. The expression of most NLRs is upregulated upon microbial infection. In contrast, the expression of NLRP12 is downregulated during VSV infection thereby allowing a robust IFN response [173]. Reduced expression upon microbial stimuli was also shown for the inhibitory protein NLRC3 [186].

In summary, the dominant inhibitory effect of NLRP12 on type I IFN induction is likely mediated by interfering with RIG-I signaling. This is in agreement with an inhibitory function of NLRP12 on NOD2 that acts in synergy with MAVS and TBK1 [174] (see also chapter on NOD2 signaling). 

### 2.8. NLRP14

The function of NLRP14 in innate immunity is hitherto not well studied. NLRP14 is expressed in the testis and ovary [219,220] and its deficiency causes reproductive failure in male and female mice [220]. Mutations in the NLRP14 gene are also associated with infertility in men [219]. Mechanistically, it was shown that NLRP14 protects HSPA2, a testis-specific HSP70 member important for germ cell differentiation and spermatogenesis, from proteasome-mediated degradation by recruiting the co-chaperone BAG2 [221]. 

Inappropriate sensing of cytosolic nucleic acids can result in auto-immune responses. The inhibition of nucleic acid sensing is particularly important during fertilization when sperm cell DNA is present in the cytoplasm of the oocyte. By mining RNA sequencing data and selecting for candidate genes specifically expressed in germ cells and downregulated after fertilization, Abe et al. identified NLRP14 as a putative nucleic acid sensing regulator [175]. In oocytes, the injection of siRNA targeting NLRP14 leads to aborted fertilization and early embryo development [222]. Abe et al. could show that the transduction of HEK293T cells with NLRP14 significantly reduced ISRE and NF-κB-mediated promoter activation in the context of STING, TRIF, TBK1, and IRF3, but not IRF7 [175]. CRISPR/Cas9 induced KO of NLRP14 in HEK293T cells stably expressing STING and siRNA knockdown in primary human bronchial epithelial cells (HBEC) resulted in enhanced DNA and RNA sensing as well as in augmented secretion of type I IFNs and antiviral immunity. Mechanistically, NLRP14 targets TBK1 for ubiquitination and degradation. Therefore, NLRP14 also impairs RIG-I- and MAVS-induced signaling. In a negative feedback loop, the adaptor molecules STING and MAVS mediate the proteasomal degradation of NLRP14, which might be important for restoring nucleic acid sensing after fertilization [175]. 

Taken together, these findings indicate that NLRP14 plays important roles in spermatogenesis and in negative control of cytosolic DNA sensing in oocytes during fertilization albeit the latter is based on a single study and awaits validation in other systems. 

## 3. Synergistic Effect of NLRs on Type I IFN Responses upon Nucleic Acid and Bacterial Sensing

### 3.1. NOD1

NOD1 is the founding member of the NLR family [108,223] and acts as a cytosolic sensor of conserved bacterial cell wall component γ-D-glutamyl-meso-diaminopimelic acid (iE-DAP) found in Gram-negative and some Gram-positive bacteria [106,110]. It is widely expressed in different cell types [108] where it is localized at membranes, mainly the plasma membranes [224,225,226]. Besides sensing of bacteria, NOD1 has also been proposed as a sensor of small GTPase activity and F-actin perturbation, caused by bacterial infections [227,228,229]. NOD1 induces NF-κB signaling via activation of its adaptor protein receptor-interacting serine/threonine-protein kinase 2 (RIPK2) [230] and is important for bacterial clearance also by interfering with autophagy [226]. 

IFN-β and poly(I:C) treatment can increase expression of NOD1 in several cell types, including adult mouse neural progenitor cells, [123]. Consequently, the transcript level of NOD1 is induced upon HCV [231], norovirus [232], and human cytomegalovirus (HCMV) infection [124]. 

NOD1 is typically wired to NF-κB activation via RIPK2, however, it can also activate IFN-β gene expression, as shown by gene reporter assays using stimulation with heat killed *Legionella pneumophila* [125]. In vivo, treatment of mice with the NOD1 ligand FK156 results in high levels of serum IFN-β, which was not observable in Nod1-deficient mice [126,127]. During *Helicobacter pylori* infection, NOD1 contributes to the epithelial induction of type I IFNs. In this process, NOD1 activation by iE-DAP can result in the interaction of RIPK2 with TRAF3, which in turn activates both TBK1 and IKKε [126]. In HT29 colon cells, stimulation with iE-DAP leads to nuclear translocation of IRF7 and IRF9, but not IRF3. Expression and nuclear translocation of the ISGF3 complex can be induced by NOD1 [126,127]. Tri-DAP stimulation of human foreskin fibroblasts has a protective effect against HCMV, but not HSV-1 infection. Tri-DAP pre-treatment increases the IFN-β response and suppresses HCMV replication, while knockdown of NOD1 results in diminished IFN-β responses [124]. NOD1 induction of IFN-β depends on RIPK2, as RIPK2 knockdown does not confer the protective effects of Tri-DAP-induced IFN-β expression during HCMV infection. Meanwhile, mutation of the CARD of NOD1 (E56K) is sufficient to blunt type I IFN secretion by disrupting the interaction with RIPK2 [124]. Interestingly, complex formation between NOD1 and dsRNA could be demonstrated by co-precipitation experiments [231]. While NOD1 can interact with MDA5 and TRAF3 in mammalian cells, fish NOD1 can directly or indirectly bind viral RNA [233]. However, as bacterial cell-wall components are widely present in surface water [234], it is well conceivable that this effect might also rely on PGN-mediated activation of NOD1.

Taken together, these data show that NOD1 is linked to type I IFN responses; however, the molecular details of this pathway remain elusive. Notably, it remains to be established what cell types can induce NOD1-dependent type I IFN responses. In human myeloid THP-1 cells for example, the NOD1-ligand fails to induce the production of the IFN-induced cytokine IP10 [126]. Moreover, in eosinophils activation of either NOD1 or NOD2 does not result in the induction of IFN-α [128]. The above discussed findings show that the effects of NOD1 on the type I IFN response are triggered by bacterial PGN activation, suggesting NOD1 as an important factor to link bacterial and viral recognition. Upon viral infection, induction of RIPK2-dependent NOD1 signaling might be of physiological importance for the higher lethality and morbidity associated with secondary bacterial infection [232].

### 3.2. NOD2

The NLR family member NOD2, which is closely related to NOD1 [235] is the cytosolic sensor for the bacterial cell wall component muramyl dipeptide (MDP) [107,111]. In contrast to the widely expressed NOD1, expression of NOD2 is more restricted to particular cell types [236,237,238]. NOD2 activates the NF-κB pathway through RIPK2 [235,239] and can induce xenophagy by recruitment of the autophagy protein ATG16L1 upon bacterial challenge [226]. Mutations in the LRR domain of NOD2 are strongly associated with Crohn’s disease (CD) [111,236,240,241]. Furthermore, NOD2 is associated with Blau syndrome [242] and atopic dermatitis [243]. As for NOD1, type I IFN-induction by NOD2 requires RIPK2 [129]. Contrary to its classically assigned role as a bacterial sensor, Sabbah and coworkers described an alternative function for NOD2 as a sensor for viral ssRNA. Mechanistically, NOD2 signals via MAVS to induce IRF3 activation and subsequent secretion of type I IFNs [130]. Activation of the NOD2–RIPK2 axis by viral RNA was also confirmed independently [244]. Moreover, NOD2 contributes to the restriction of HCMV via induction of type I interferons [131]. Besides activation by viruses, NOD2 can also augment type I IFN responses upon bacterial challenges. Here, sensing of bacterial lysosomal degradation products by NOD2 results in a signaling cascade that induces transcription of type I IFNs by IRF5 [129,132,133,134] in synergy with other PRR signaling pathways [245]. While MDP stimulation alone does not result in NOD2-dependent activation of an IFN-β reporter gene, foot and mouth disease virus-induced IFN-β and ISG15 transcription is reduced by siRNA-mediated knockdown of NOD2 [135], suggesting a synergistic effect. This has also been documented in the colon of mice, where NOD2 drives a type I IFN response that instigates microbial colonization resistance. This process is then timely regulated by NLRP12 which targets NOD2 for degradation. [174]. However, the role of NOD2 in the induction of IFN-β responses towards bacterial pathogens appears to be dependent on the bacterial species, as IFN-β responses were not impaired in *Nod2*^−/−^ mice after *Streptococcus pneumoniae* and *L. monocytogenes* challenges [246,247]. 

While a beneficial role of NOD2, was reported during IAV infection, this was only observed after additional challenge with MDP [248]. This argues for a synergistic effect of MDP-induced NOD2 signaling and viral sensing. Accordingly, knockout of MAVS abolishes this positive effect, indicating a dependency on RIG-I signaling [248]. Knockdown of either NOD2 or MAVS also abolishes the effects of leukotriene B4 treatment on IAV infection in mice [249]. Whether NOD2-induced activation of RIPK2 differs between MDP sensing, or binding of viral ssRNA, remains to be elucidated. 

In contrast to the proposed positive role of NOD2 in antiviral defense, negative crosstalk between RIG-I and NOD2 was postulated by Morosky et al. They showed interaction of overexpressed RIG-I and NOD2. While RIG-I inhibits NOD2 induced NF-κB activation, a dose-dependent negative influence of NOD2 on RIG-I induced IFN-β promoter activity was observed [136]. This was also shown for zebrafish RIG-I and NOD2 [250]. The underlaying mechanisms of this mutual negative regulation remains unclear but may depend on the expression of some cell-type specific adaptor protein. Indeed, K63-polyubiquitination of TRAF6 was negatively regulated by MDP-induced IRF4 expression in monocyte-derived cells [251,252,253]. 

NOD2 has long been thought of as a general bacterial sensor. It recently has become evident that NOD2 also contributes to viral sensing. It remains to be fully established, whether the effect of NOD2 in the induction of IFN responses is direct or indirect, by activation of the NF-κB pathway and interplay with RIG-I. Recent advances however suggest that effects are rather synergistic and, at least in part induced by activation of NOD2 by bacterial PGN. This nicely illustrates the intimate interplay between bacterial and viral sensing and has important implications in infectious diseases and auto-inflammatory disorders. 

### 3.3. NLRC4

NLRC4 can also form an inflammasome even if being atypical, as it does not contain a PYD. Sensing of the bacterial activators flagellin [254,255,256] and the rod protein of type 3 secretion systems [112] by the NLRC4 inflammasome is not performed by the protein itself. Instead, it relies on the NAIP family of proteins. NAIP expression is regulated by IFN regulated factor 8 (IRF8), which is directly regulated by type I IFN signaling [257,258]. Accordingly, loss of Irf8 lowers the expression of *Nlrc4* and Nlrc4 inflammasome activity in mouse BMDMs [258]. Hence, the function of the NLRC4 inflammasome can be influenced by type I IFNs, albeit the authors found that Irf8 regulated *Naip* expression independently of IFN signaling [258].

In mice, *Naip5* was initially identified as a susceptibility marker for *Legionella pneumophila*. Susceptibility for Legionella infection is mimicked by deletion of Irf1 and Irf8, respectively. Elegant genetic studies revealed a strong genetic interaction between Irf8 and Nlrc4, suggesting that the NLRC4 inflammasome itself might induce type I IFN responses by Irf8 [150]. In line with the above discussed function of NOD1 and NOD2 in linking bacterial sensing to antiviral responses, in DCs, activation of NLRC4 by bacterial flagellin and subsequent secretion of IL-22 and IL-1β results in better control of rotavirus infection [259]. 

### 3.4. NLRP6

NLRP6 forms an inflammasome involved in sensing of Gram-positive bacterial pathogens and viruses. Upon activation, it can modulate innate and adaptive immunity by triggering IL-1β and IL-18 production [260]. In terms of a link to type I IFN responses, it was shown that NLRP6 expression can be upregulated by IFN signaling induced by lipoteichoic acid from *L. monocytogenes* that also serves as an activator of the NLRP6 inflammasome [167]. 

Wang et al. showed that NLRP6 contributes to control encephalomyocarditis virus (EMCV) infection and norovirus infection [168]. Functionally, NLRP6 interacts with the RNA-helicase Dhx15 to form a sensor for viral dsRNA that subsequently recruits MAVS for the induction of antiviral type I and III IFN responses [168]. It will be interesting to elucidate whether Dhx15 has specificity for particular viral RNAs. By contrast, LPS stimulation results in temporarily enhanced type I IFN transcription in NLRP6 knock-out mice [261].

At present, more work is needed to elucidate the physiological role of NLRP6 in type I IFN responses. The formation of a complex of an NLR with a DEAD-box helicase as sensor for ribonucleic acids, however, might be a common theme as Nlrp9b also uses the DEAD-box protein Dhx9 as a sensor for double-stranded RNA [169], and sensing of viral and bacterial RNA by DHX33 has been shown to enhance NLRP3 inflammasome formation [262] (see below).

### 3.5. NLRP9

NLRP9 is highly expressed in the reproductive system [205], where it can form an inflammasome [263,264]. While only one *NLRP9* gene is present in humans, there are duplications of this gene, leading to three paralogs (*Nlrp9a*, *Nlrp9b*, and *Nlrp9c*) in rodents [209]. The mouse Nlrp9b protein is expressed in intestinal epithelial cells and contributes to restriction of rotavirus infection by inducing pyroptotic cell death. This is mediated by complex formation with Dhx9, an RNA-helicase that recognizes short dsRNA and mediates inflammasome formation by recruitment of ASC [169]. However, this function is not shared by the other Nlrp9 paralogs in mice. It thus remains to be established how human NLRP9 contributes to type I IFN responses. 

## 4. Type I IFN Modulates the Quality of the NLR Response to Infection

### NLRP3

NLRP3 is one of the best characterized NLR proteins. It is a cytosolic, inflammasome-forming NLR [265,266]. Assembly of the NLRP3 inflammasome takes place at the perinuclear centrosome, the microtubule-organizing center (MTOC) of the cell [267]. Before assembly of the NLRP3 inflammasome, a priming step is required. This priming step is thought to function through different mechanisms. NLRP3 expression is upregulated in an NF-κB-dependent manner by TLR signaling and post-translational modifications as deubiquitination and phosphorylation were also reported to play a role in the priming step [268,269,270]. 

Besides the classical priming of NLRP3 expression by NF-κB-dependent mechanisms, it was suggested that the expression of NLRP3 is also regulated by type I IFNs. Indeed, knockdown of IFN-β secretion reduces NLRP3 transcription and activity [160], while pathogen-induced type I IFN triggers activation of the non-canonical inflammasome pathway via up-regulation of caspase-11 [161,162]. On the contrary, other studies report an inhibition of the NLRP3 inflammasome upon type I IFN signaling. The Nlrp3 inflammasome might be inhibited by type I IFNs in two ways. First, in vitro IFN-β treatment results in a decrease of caspase-1 activation and reduced levels of both pro-IL-1β and IL-1β. Both type I and type II IFNs induce the expression of IFN gamma inducible protein 16 (IFI16), which is a negative regulator of the NLRP3 inflammasome [271]. Second, IFN-β results in an enhanced secretion of IL-10, which in turn reduces the levels of pro-IL-1β [272]. In mice, it was reported that inhibition of the Nlrp3-coupled inflammasome by IFN-β is dependent on NO, possibly mediated through thiol nitrosylation of NLRP3 [273]. 

In term of a function in antiviral responses, Nlrp3 protects mice against some RNA viruses (IAV and human rhinovirus) via the regulation of caspase-1 [274,275,276]. Activation of NLRP3 by viral pore forming proteins (viroporins) such as the IVA M2 protein or coronavirus viroporin 3a has been proposed as activating mechanism [275,277,278]. NLRP3 might further act as a sensor of dsRNA [276]. In these processes, MAVS has been implicated in ubiquitination of ASC leading to enhanced NLRP3 inflammasome activity by its recruitment to mitochondria [279,280].

## 5. NLR-Mediated Diseases Join the Expanding Spectrum of Interferonopathies

The generic term of ‘type I interferonopathy’ has been conceptualized based on clinical evidence of biological similarities between the sickness behaviors of several auto-inflammatory conditions with a constitutive upregulation of type I IFN production, including Aicardi-Goutières syndrome [281,282]. Failure to properly induce or maintain type I IFN responses towards either infections or tissue injury goes hand in hand with increased pathogenicity and morbidity of infectious and auto-inflammatory diseases [283,284]. This is reflected by the fact that many viruses have evolved evasion mechanisms targeting antiviral IFN responses. Type I IFNs have found their way into the clinic and are used as drugs to combat viral infection. For treatment of hepatitis B and hepatitis C virus infections, IFN-α was being used in conjunction with antiviral therapy, and they are experimentally tested for other viral diseases [285,286]. Advances in drug development and treatment regimens for viral hepatitis, however, have led to the replacement of the use of IFN by more specific and antiviral regimens with less side effects [287]. Despite a little contribution from adaptive immunity, it has become clear that the range of interferonopathies is broader than previously thought. The advent of exome sequencing revealed that mutations in genes encoding several components of the antiviral IFN response can also cause interferonopathies. The study of the molecular drivers of this disease helped the identification of the cytosolic nucleic acid sensing system. Overshooting type I IFN responses are highly detrimental to the host and can either exacerbate the disease burden after viral infection, or even lead to a lowered tolerance of the tissue to the damage caused by viruses [288,289]. It is hence crucial that type I IFN responses are precisely regulated and can be downregulated at an appropriate time. Whether a type I IFN response will be beneficial or detrimental in many cases depends on timing and intensity, as well as on proper control and reduction at an appropriate time. Moreover, due to their action on the adaptive immune system, beta IFNs are used for treatment of the auto-immune disease multiple sclerosis (MS) [290]. 

Several of the above discussed NLR proteins have been identified as factors either involved in or driving such pathologies. As described in detail above, a negative regulatory effect of IFN-β on NLRP3 inflammasome formation and activation has been shown in several studies. An involvement of the NLRP3 inflammasome in IFN-β therapy of MS patients was hence suggested [272,291]. In primary human monocytes upon being treated with IFN-β, both NLRP3-induced IL-1 β secretion and caspase-1 activation are decreased [272]. In the mouse experimental auto-immune encephalomyelitis (EAE) model, modelling MS, the mechanism behind effective IFN-β treatment, was shown to be conferred through IFN-receptor-mediated induction of Socs1, which further attenuates activation of Rac1 and decreases the production of radical oxygen species (ROS). This in turn is thought to result in the inhibition of the NLRP3 inflammasome. This group also reported that IFN-β therapy is not effective when EAE is induced in an NLRP3-independent manner [292]. An association of NLRs with auto-inflammatory diseases is also the case for the dysregulation of the NLRC4 inflammasome in macrophage activation syndrome, a disease which is characterized by highly aberrant levels of IL-18 and IFN-γ [293]. Thus, NLR-mediated control of type I IFN responses also applies to the adaptive arm of the immune system. Mutations of NLRP12 are associated with FCAS, which is characterized by intermittent episodes of fever and serosal inflammation [218]. *Nlrp12*^−/−^ mice are highly susceptible to colitis and colitis-associated colorectal cancer [215,294], whereas they are resistant to Salmonellosis [216]. Normand et al. revealed a novel function of NLRP12 as a monocytic checkpoint blocker for NOD2 signaling [174]. They could show that NLRP12 interacts with NOD2 and promotes K48-linked polyubiquitination and degradation of NOD2 in response to MDP. This leads to subsequent inhibition of the canonical NF-κB pathway and represses the activity of the JAK/STAT signaling pathway. The FCAS causing NLRP12 mutation R284X fails to inhibit NOD2-dependent NF-κB and TBK1 activation. *Nlrp12*^-/-^ mice showed significant overexpression of ISGs in the caecum including IFN-induced protein 44 (IFI44), IFN-induced protein with tetratricopeptide repeats 2 (IFIT2), apolipoprotein L9 as well as 2′-5′-oligoadenylate synthetase 2 (OAS2) and represented with an enhanced activation of STAT1 in the caecum, which might influence the persistence of some enteric viruses, such as endemic noroviruses [174]. Furthermore, a rare genetic variant of the NLRP14-encoding gene that confers reduced suppression of TBK1 activity is present at a frequency of about 1.7% worldwide [175]. Unphysiologically high levels of type I IFN disrupt the formation of seminiferous tubules resulting in the loss of germ cells [295] and the intraperitoneal injection of IFN-α leads to decreased spermatogenesis [296]. In line with these observations high levels of IFN-α were measured in infertile men [297], alluding to the above discussed function of NLRs to control IFN responses in the reproductive system. 

## 6. Conclusions

A functional and well-balanced type I IFN response is fundamental to many immunological processes. Failure to either induce, maintain, or properly control type I IFN responses results in impaired control of infection and immunopathology at the extreme ends and perturbations in type I IFN responses are associated with dysregulated immune balance in the innate and adaptive arm. 

Members of the NLR-family of proteins recently emerged as regulators of type I IFN responses and can negatively and positively contribute to signaling outcome (Figure 2, Table 1). Moreover, some NLRs can act as direct or indirect sensors of viruses or can induce type I IFN signaling towards infectious challenge. Complex formation with DEAD-box helicases thereby emerges as a general mechanism for indirect sensing of viral nucleic acids, exemplified by Nlrp9b and NLRP6. It remains to be established if and which DEAD-box helicases may also account for the sensing of viral RNA by either NOD2, NLRC4, and NLRP12. 

The above discussed examples reveal that the aforementioned NLRs fundamentally contribute to link bacterial sensing and antiviral immunity. This has not only to be considered in the context of bacterial infection, but also applies to the tolerance towards the viral component of the microbiota. The role of type I IFNs as negative regulators of IL-1β production, which is mediated by NLR-containing inflammasomes, is becoming increasingly evident [272,291,298,299]. Moreover, it is known that type I interferons can induce anti-inflammatory cytokines (such as IL-10) and thereby can repress the expression of pro-inflammatory cytokines and chemokines as well as of anti-microbial peptides, and by this contribute to the quality of anti-bacterial responses [300]. The role of NLRs in this context is only emerging, and it will be interesting to address this in more detail in future work.

The molecular details of the function of most NLRs in the negative regulation of type I IFN responses are not fully defined yet. However, from the current advances, at least two general mechanisms of NLR functions in type I IFN signaling emerge: (i) Hindrance of interaction of signaling adaptors (such as TRAFs and MAVS) by physical interaction with NLRs, and (ii) activation of E3-ligases by NLRs that leads to proteosomal degradation of key components of IFN signaling (such as TBK1). For this degradation, autophagy might be another central hijacking hub [301], which should be address in future work.

Future studies will help to gain more knowledge about the underlying mechanisms of how NLRs act in type I IFN responses and will lead to a to better understanding of the role of NLRs in infectious and auto-inflammatory disease. In the long run, this will help to define novel targets for better therapeutic intervention. For the latter, the link of bacterial activation of NLRs and antiviral responses seems to be a promising avenue when considering target intervention of the gut microbiome.

## Figures and Tables

**Table 1:** Role of Nod-like receptors (NLRs) in interferon responses. The upward arrow indicates induction.

Name	Activator/Ligand	Induction by Type I IFN	Function in Type I IFN Response
NOD1	iE-DAP	↑ [123]	Positive regulation of type I interferon responses [124,125,126,127,128]
NOD2	MDP; viral ssRNA	Unknown	Positive regulation of type I interferon responses [128,129,130,131,132,133,134,135,136,137]
NLRX1	Unidentified	Unknown	Negative regulation of RIG-I/MAVS- and STING-dependent type I interferon response [105,138,139,140,141,142,143,144,145,146,147,148]
NLRC3	Unidentified	Unknown	Negative regulation of type I interferon response by disruption of proper STING trafficking [149]
NLRC4	NAIP	Unknown	Enhanced IFN response by Irf8 [150]
NLRC5	Unidentified	Unknown	Induction of MHC class I expression [114,115,151] Regulation of type I interferon responses but controversial data [114,115,143,151,152,153,154,155,156]
NLRP2	Unidentified	↑ [157,158]	Negative regulation of IFN-β response by inhibiting TBK1 [159]
NLRP3	ROS; membrane rupture; cathepsins; K+ efflux; dsRNA	↑ [160,161,162]	No known functions in regulation of IFN response
NLRP4	Unidentified	Unknown	Negative regulation of type I interferon response through degradation of TBK1 by recruitment of DTX4 [163,164,165,166]
NLRP6	lipoteichoic acid	↑ [167]	Positive regulation of type I and III IFN responses by interaction with Dhx15 to form a sensor for dsRNA [168]
NLRP9	Unidentified	Unknown	Nlrp9b: Restriction of rotavirus infection by complex formation with Dhx9 which recognizes short dsRNA [169]
NLRP11	Unidentified	Unknown	Negative regulation of type I interferon response [170,171,172]
NLRP12	Unidentified	Unknown	Inhibition of type I interferon response via the degradation of RIG-I and NOD2 [173,174]
NLRP14	Unidentified	Unknown	Negative regulation of type I interferon response through degradation of TBK1 [175]
